# Adoptively transferred melanoma-reactive CTL primed in the presence of IL-21 and transferred with concurrent CTLA4 blockade results in enhanced CTL persistence and tumor regression

**DOI:** 10.1186/2051-1426-2-S1-O2

**Published:** 2014-02-24

**Authors:** Cassian Yee, Aude Chapuis, Phil Greenberg, Ivy Lai, John A Thompson, Kim Margolin

**Affiliations:** 1 Melanoma Medical Oncology, MD Anderson Cancer Center, Houston, Texas, USA; 2 Clinical Research Division, Fred Hutchinson Cancer Research Center, Seattle, Washington, USA

## 

Anti-CTLA4 monotherapy yields long-term benefit in < 10% of patients with metastatic melanoma, with most patients failing to respond likely due to inadequate expansion of endogenous tumor-reactive T cells. We postulated that adoptive transfer of melanoma-reactive CD8+ cytotoxic T lymphocytes (CTL) primed in the presence of Interleukin-21 (IL-21), which programs antigen-specific cells to retain characteristics of long-lived memory cells, could provide a bolus of immune effectors with enhanced in vivo persistence that, in combination with anti-CTLA4 blockade, would lead to tumor regression. Of ten patients with therapy-resistant melanoma, 4 patients experienced durable clinical responses, including 2 complete responders (CR,100% regression), and 2 partial responders (PR, 79% and 75% regression respectively). Three additional patients experienced stable disease (SD). The infused CTL were detected in the peripheral blood at frequencies up to 8% in all patients for up to 40 weeks. In patients with clinical benefit (CR, PR, SD), the persisting CTL exhibited CD28 expression, secreted IL-2, proliferated in vitro, and acquired central memory phenotype. These patients developed de novo anti-tumor responses (epitope spreading) to non-targeted antigens. This study represents a first-in-man study combining antigen-specific T cell therapy with immune checkpoint blockade, and highlights a potential synergistic biologic and therapeutic effect.

